# Synchronous photocatalytic benzimidazole formation and olefin reduction by magnetic separable visible-light-driven Pd-g-C_3_N_4_-Vanillin@γ-Fe_2_O_3_-TiO_2_ Nanocomposite

**DOI:** 10.1038/s41598-024-76198-z

**Published:** 2024-11-06

**Authors:** Maasoumeh Jafarpour, Abdolreza Rezaeifard, Narges Pourmorteza, Maryam Ghanbari Kudeyani

**Affiliations:** 1 Department of Organic Chemistry, Faculty of Chemistry and Petroleum Sciences, Bu-Ali Sina University, Hamedan, 65178-38695 Iran; 2 Department of Inorganic Chemistry, Faculty of Chemistry and Petroleum Sciences, Bu-Ali Sina University, Hamedan, 65178-38695 Iran; 3 Catalysis Research Laboratory, Department of Chemistry, Faculty of Science, University of Birjand, Birjand, 97179-414 Iran; 4 South Raadab Engineering Company, Zahedan Desalination Plant, Zahedan, 98169-13419 Iran

**Keywords:** Photocatalysts, Vanillin, Benzimidazole, Olefin reduction, Nanocomposite, Chemistry, Catalysis, Photocatalysis

## Abstract

A visible light-responsive magnetically separable photocatalyst Pd-g-C_3_N_4_-Vanillin@γ-Fe_2_O_3_-TiO_2_ is fabricated using vanillin as a natural junction under ultrasonic agitation. Structural, morphological, optical, thermal, and magnetic assessments of the as-prepared catalyst are carried out. The photocatalyst successfully drives the simultaneous benzimidazole formation, and olefin hydrogenation with high atom economy under blue LED light and mild conditions. The photocatalytic activity of Pd-g-C_3_N_4_-Vanillin@γ-Fe_2_O_3_-TiO_2_ is significantly affected by the γ-Fe_2_O_3_:TiO_2_ weight ratio. PL spectra revealed the effective separation of carriers in the fabricated catalyst promoting its photocatalytic activity. The action spectra using the apparent quantum efficiency (AQE) exhibited the maximum AQEs at 520 and 750 nm in which the highest performance for styrene hydrogenation is observed. The title magnetically separable heterogeneous photocatalyst provides high yields of products under perfectly safe visible light, produces low/zero waste, and avoids using an external high-pressure hydrogen gas source, harmful solvents, undesirable additives, and reducing agents rendering green conditions for chemical reactions.

**Supplementary Information:**

The online version contains supplementary material available at 10.1038/s41598-024-76198-z.

## Introduction

Semiconductor photocatalysis is becoming one of the most active research areas and has been studied in different streams such as catalysis; photochemistry; electrochemistry; inorganic and organic chemistries; physical, and polymer, environmental chemistry, and so on. Mainly binary semiconductors such as TiO_2_, ZnO, Fe_2_O_3_, CdS, and ZnS have been used as photocatalysts because of a favorable combination of their electronic structure, light absorption properties, charge transport characteristics, and excited-state lifetime^1,2^. Considering the benefits and limitations of these photocatalytic materials, some researchers have attempted to enhance the photocatalytic activity of these materials using various techniques. Different strategies have been used from time to time, such as surface and interface modification by controlling morphology and particle size, composite or coupling materials, transition metal doping, nonmetal doping, codoping (metal– metal, metal–nonmetal, nonmetal–nonmetal), noble metal deposition, and surface sensitization by organic dye and metal complexes, to enhance the photocatalytic properties^3–6^.

Among the large family of semiconductors, magnetic metal oxides, which possess magnetic and photonic properties that provide the requirements for constructing an easily separable photocatalyst, are of great interest. The chemically stable and earth-abundant ferric oxide (Fe_2_O_3_), a conventional *n*-type semiconductor with a band gap of 2.2 eV, is currently considered one of the most promising materials for solar energy conversion and consequently photocatalytic processes^7,8^. But low conductivity, higher e^−^–h^+^ recombination effect, a short length of holes diffusion, and the low conduction band position in Fe_2_O_3_ impede its practical applications^9–11^. Therefore, the development of novel photocatalysts with higher quantum efficiency and low recombination of photogenerated charges has become a challenging topic in the magnetic photocatalytic field^12^. One of the promising approaches to overcome this disadvantage is coupling Fe_2_O_3_ with other semiconductors promoting the light-harvesting ability and optical properties caused by the charge separation in the visible light spectrum. Furthermore, the coupling of narrow band gap Fe_2_O_3_ with wide bandgap semiconductors such as ZnS, SnO_2_, and TiO_2_ forms magnetic composite materials with enhanced visible light response^13–17^. This nature of magnetic photocatalyst could accelerate the separation rate of charge carriers in photocatalytic applications, especially for coupling with TiO_2_ nanocrystals because of its well anti-photo corrosion properties. Compared to conventional nanopowder photocatalysts, TiO_2_ magnetic composites such as Fe_2_O_3_–TiO_2_^18–20^, or Fe_3_O_4_–TiO_2_^21^, can be regarded as promising photocatalysts for environmental purification at the industrial scale as they can be more readily separated from the slurry system by the magnetic separation after photocatalytic reaction and recycled^22–24^.

Graphitic carbon nitride (g-C_3_N_4_) as a metal-free photocatalyst with a bandgap of 2.7 eV capable of visible-light absorption^25^, with high thermal and chemical stability, is especially suitable for applications in photochemistry and photocatalysis. However, a fast recombination rate of carriers (electron-hole) and low visible light absorption range reduce its photocatalytic conversion efficiency which greatly limits its practical applications^26^. Various modification strategies including synthesizing mesoporous structures^27,28^ or nanocomposite structures^29,30^, introducing heteroatoms^31–34^, copolymerization^35^, coupling with dyes^36^ or other semiconductors^37,38^, etc. have been devoted to overcome these problems and improve its photocatalytic efficiency. Among these, post-grafting strategy based on Schiff base chemistry has been developed to fabricate novel aromatic-grafted carbon nitrides^39^. The post-grafting of aromatic rings into the g-C_3_N_4_ network did not disrupt the original framework of g-C_3_N_4_, but effectively expanded its п-delocalized system, enlarged its surface area, and promoted the separation and transfer of photo-excited charge carriers.

This strategy is general and can be used to graft aromatic rings of different molecular structures onto g-C_3_N_4_. With these outstanding features in mind, we have successfully developed a novel g-C_3_N_4_-based composite photocatalyst based on the Schiff base interaction of the aldehyde group of vanillin and surface-exposed terminal amino groups in g-C_3_N_4_.

The catalytic reduction of olefins to valuable products is a reaction of major importance in organic synthesis^40–45^. Metal-catalyzed hydrogenations without a doubt represent a powerful and practical method for the reduction of olefins to produce the corresponding saturated hydrocarbons, which are important feedstock for the petrochemical industry^46^. This transformation can be realized by heterogeneous catalysts containing noble metals such as Au, Pt, and Rh with flammable hydrogen gas as a reducing agent. Although, H_2_-hydrogenation is an atom-efficient reaction, the use of high-pressure flammable molecular hydrogen limits seriously its practical applications^47,48^. The transfer hydrogenation reaction (TH) that replaces H_2_ gas with inexpensive hydrogen donors such as NaBH_4_, N_2_H_4_.H_2_O, formic acid, and isopropanol has received more attention^49–51^. However, some limitations such as the use of stoichiometric amounts of reagents, harsh reaction conditions, substrate scope, recyclability of the catalyst, and low efficiency restricted seriously the use of common hydrogen donors^52^. Thus, performing the hydrogenation reaction using in situ-generated H_2_ (dehydrogenation/hydrogenation process) is in extreme demand.

Just recently, we discovered an unprecedented visible-light-driven photocatalytic system consisting of Pd nanoparticles stabilized on g-C_3_N_4_-imine-functionalized TiO_2_ nanoparticles (Pd-gC_3_N_4_Imine/TiO_2_) for photoassisted hydrogen generation followed by olefin hydrogenation under mild conditions. Several types of transformations in a sequential one-pot strategy were promoted including synchronous photocatalytic production of hydrogen and acceptorless synthesis of benzimidazoles followed by the photocatalytic olefins hydrogenation under mild reaction conditions.^53^

In this work we have made two modifications in the structure of the previously reported photocatalyst to achieve many benefits: (1) replacement of TiO_2_ nanoparticles (NPs) with magnetic counterpart (γ-Fe_2_O_3_-TiO_2_ NPs) enables the photocatalyst to be separated easily by an external magnet; (2) replacement of commercially unavailable and unsafe (3-oxopropyl) trimethoxysilan with vanillin as a natural and safe product to join the g-C_3_N_4_ to γ-Fe_2_O_3_-TiO_2_ meanwhile, the easy condensation of g-C_3_N_4_ with vanillin under microwave irradiation to produce g-C_3_N_4_-vanillin Schiff base is a further advantage for replacing the present photocatalyst than the previous one. The as-prepared magnetic nanohybrid Pd-g-C_3_N_4_-vanillin@γ-Fe_2_O_3_-TiO_2_ successfully derived several invaluable processes. The in situ H_2_ generation from an acceptorless dehydrogenation of benzimidazoline intermediate followed by the hydrogenation of olefins under visible-light and mild reaction conditions to produce biologically important benzimidazoles alongside saturated hydrocarbons. The γ-Fe_2_O_3_:TiO_2_ weight ratio was shown to play a key role in improving the photocatalytic performance of the as-prepared Pd-g-C_3_N_4_-vanillin@γ-Fe_2_O_3_-TiO_2_ for hydrogenation of olefins. The higher olefin hydrogenation performance of Pd-g-C_3_N_4_-vanillin@γ-Fe_2_O_3_-TiO_2_ than the nonmagnetic and vanillin-free Pd-gC_3_N_4_Imine/TiO_2_ photocatalyst is another advantage of the present photocatalyst. Meanwhile, the magnetically separable photocatalyst is air-stable, robust, recyclable, and very active under visible light and mild conditions in the absence of any undesirable additives, and reducing agents which makes it amenable to large-scale applied goals.

## Results and discussion

### Catalyst fabrication and structural analysis

**Fig. 1:**
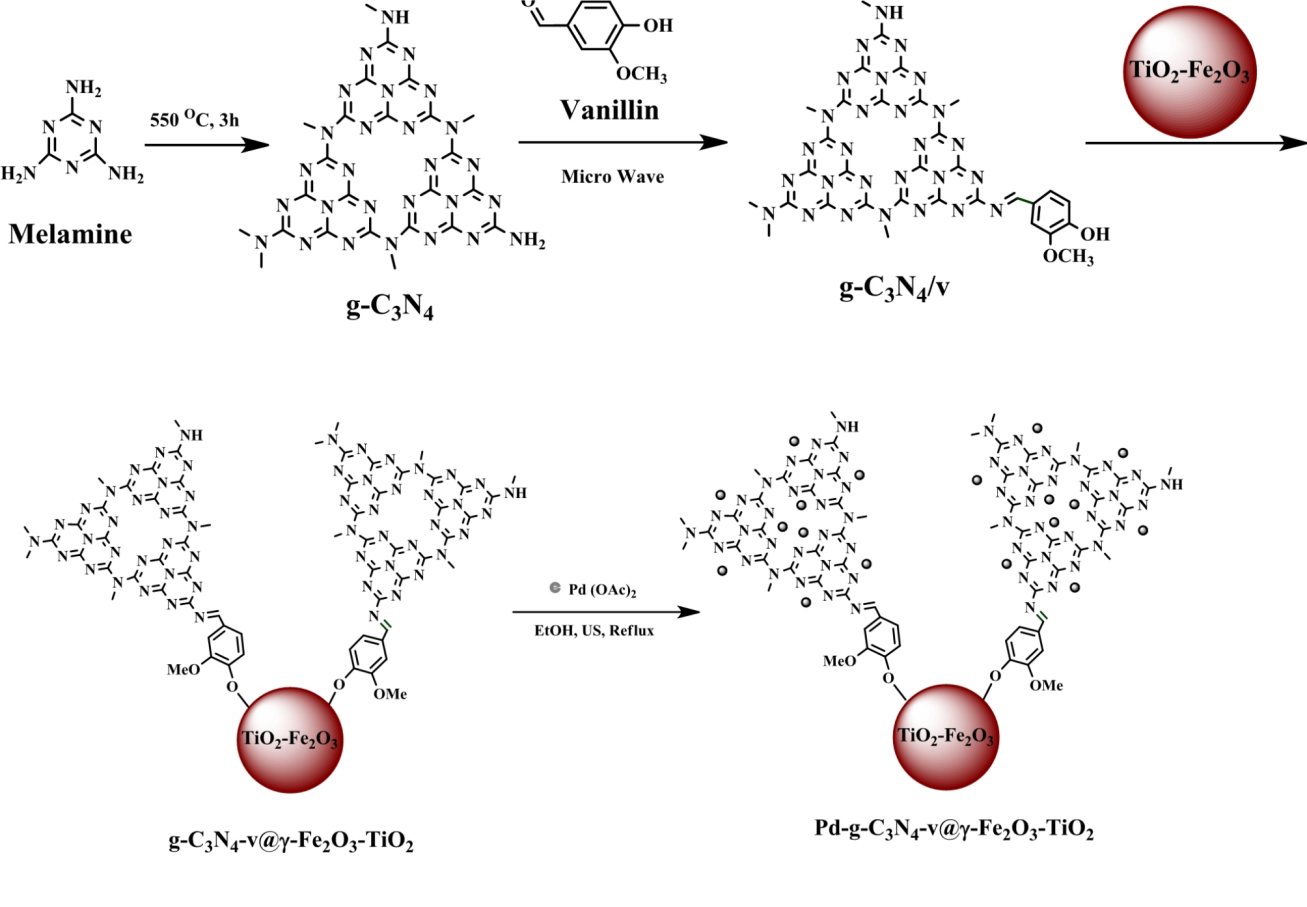
Synthetic route for Pd- g-C_3_N_4_-v@γ-Fe_2_O_3_-TiO_2_ nanocatalyst.

 The multi-step synthesis of the catalyst is depicted in Fig. 1. γ-Fe_2_O_3_-TiO_2_^54^ and g-C_3_N_4_^53^, were synthesized according to the reported procedures with minor modifications. Condensation of the − NH_2_ group of g-C_3_N_4_ with vanillin (v) under microwave irradiation (100-W) produced a new compound of g-C_3_N_4_/v prone to attach to γ-Fe_2_O_3_-TiO_2_ to produce the g-C_3_N_4_-v@γ-Fe_2_O_3_-TiO_2_ nanocomposite as a catalyst precursor. Finally, the desired Pd-containing catalyst (Pd-g-C_3_N_4_-v@γ-Fe_2_O_3_-TiO_2_) nanocomposite was prepared by the addition of Pd(OAc)_2_ to g-C_3_N_4_-v@γ-Fe_2_O_3_-TiO_2_ nanocomposite under ultrasonic agitation (Fig. 1).

**Fig. 2:**
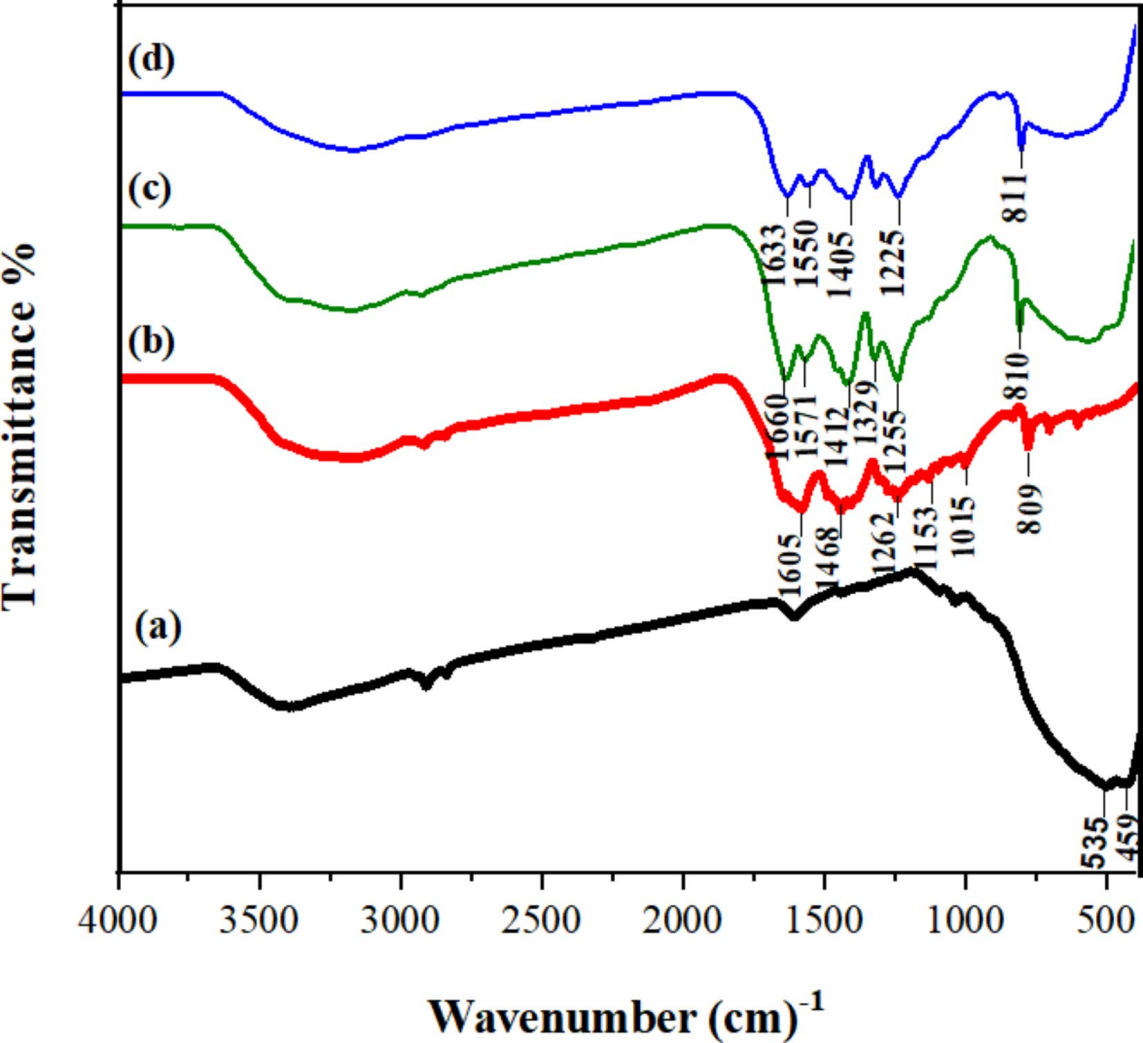
FT-IR spectra of (**a**) γ-Fe_2_O_3_-TiO_2_ (**b**) g-C_3_N_4_-v (**c**) g-C_3_N_4_-v@γ-Fe_2_O_3_-TiO_2_ (**d**) Pd-g-C_3_N_4_-v@γ-Fe_2_O_3_-TiO_2_ nanohybrid.

The FT-IR analysis is used to detect the chemical compositions and bonding information of the as-prepared samples and the results are shown in Fig. 2. The strong and broad bands at about 459–535 cm^− 1^ in Fig. 2a, c, d are assigned to the vibration modes of Fe–O bonds in the γ-Fe_2_O_3_-TiO_2_ nanoparticles and a slight shift towards lower wavenumber in Fig. 2c and d can be caused by coordination of γ-Fe_2_O_3_-TiO_2_ to g-C_3_N_4_-Vanillin hybrid and Pd(II)center. The typical band of TiO_2_ at 450–750 cm^− 1^ assigned to the stretching vibrations of Ti–O groups can be clearly observed in Fig. 2a, c, d^55^. The broad peak at 3400 cm^− 1^ originated from O-H bands and surface adsorbed water of **γ-**Fe_2_O_3_-TiO_2_ particles^56^. In the FT-IR spectrum of g-C_3_N_4_-v (Fig. 2b), bands located at 1015 and 1605 cm^− 1^ correspond to typical stretching modes of the C–N and C = C stretching vibration in the aromatic ring, respectively. While the sharp peak centered at 809 cm^− 1^ is attributed to the typical tri-s-triazine derivatives of g-C_3_N_4_^57^. The emergence of characteristic peaks of components g-C_3_N_4_ and γ-Fe_2_O_3_-TiO_2_ in Fig. 2c, d, confirmed the successful construction of the as-prepared composites.

**Fig. 3:**
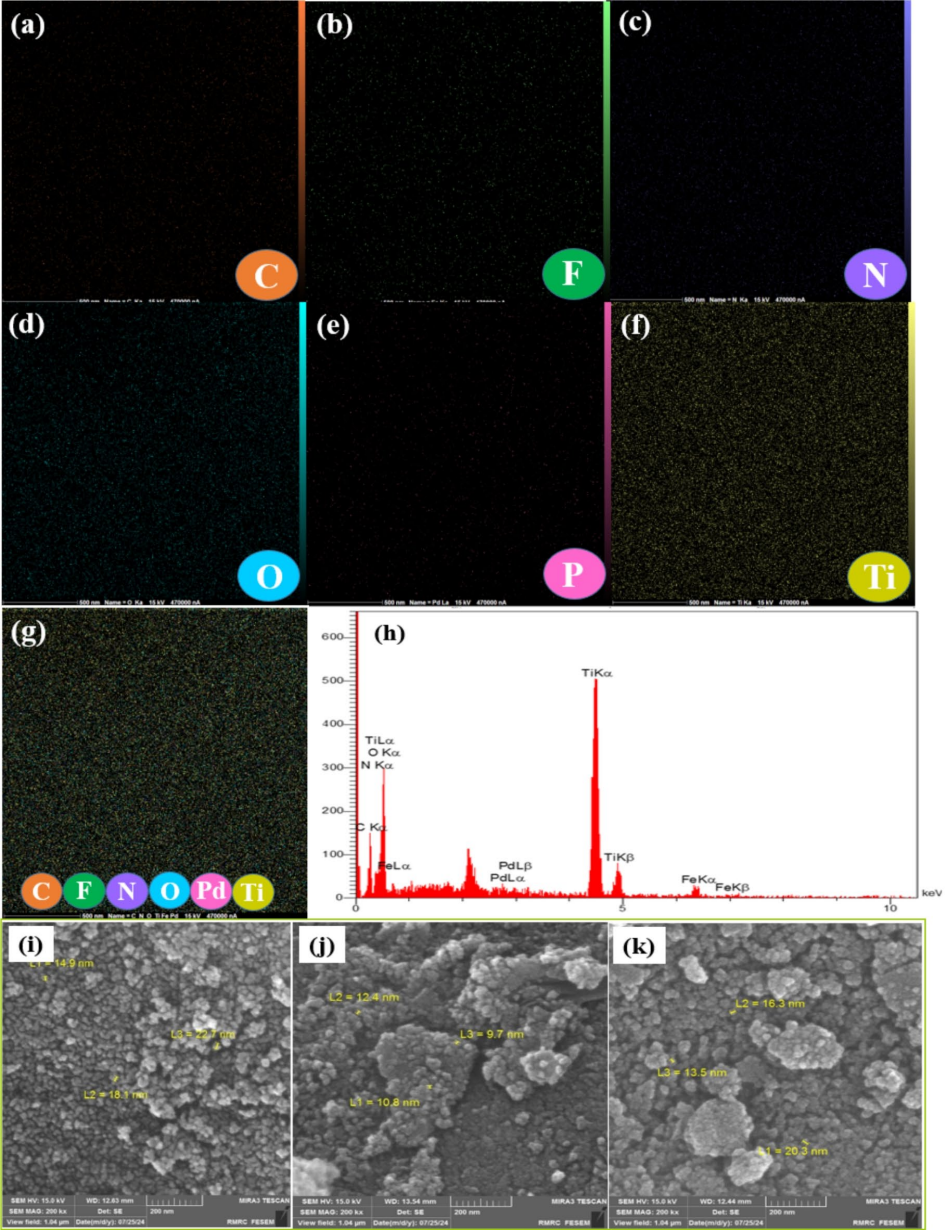
Elemental mapping images (**a**-**g**); EDX analysis (**h**) FESEM image of (**i**) γ-Fe_2_O_3_-TiO_2_, (**j**) g-C_3_N_4_-v@γ-Fe_2_O_3_-TiO_2_, (**k**) Pd-g-C_3_N_4_-v@ γ-Fe_2_O_3_-TiO_2_ nanohybrid.

Elemental mapping at the microstructural level by FE-SEM with EDS spectrum of Pd- g-C_3_N_4_-v@γ-Fe_2_O_3_-TiO_2_ nanocatalyst is shown in Fig. 3a-k which confirmed the presence of Ti, C, Fe, O, N, and Pd in the heterostructure. FE-SEM images of γ-Fe_2_O_3_-TiO_2_, g-C_3_N_4_-v@γ-Fe_2_O_3_-TiO_2_, and Pd-g-C_3_N_4_-v@ γ-Fe_2_O_3_-TiO_2_ nanohybrid are given in Fig. 3i-k respectively. A spherical morphology was found with increased agglomeration after decoration with organic parts and Pd indicating successful modification of γ-Fe_2_O_3_-TiO_2_ with Pd-g-C_3_N_4_-v. An average size of 13–20 nm was found for the Pd-g-C_3_N_4_-v@γ-Fe_2_O_3_-TiO_2_ nanocatalyst. The precise palladium content was found to be 0.10 mmolg^− 1^ based on the ICP-OES analysis.

**Fig. 4:**
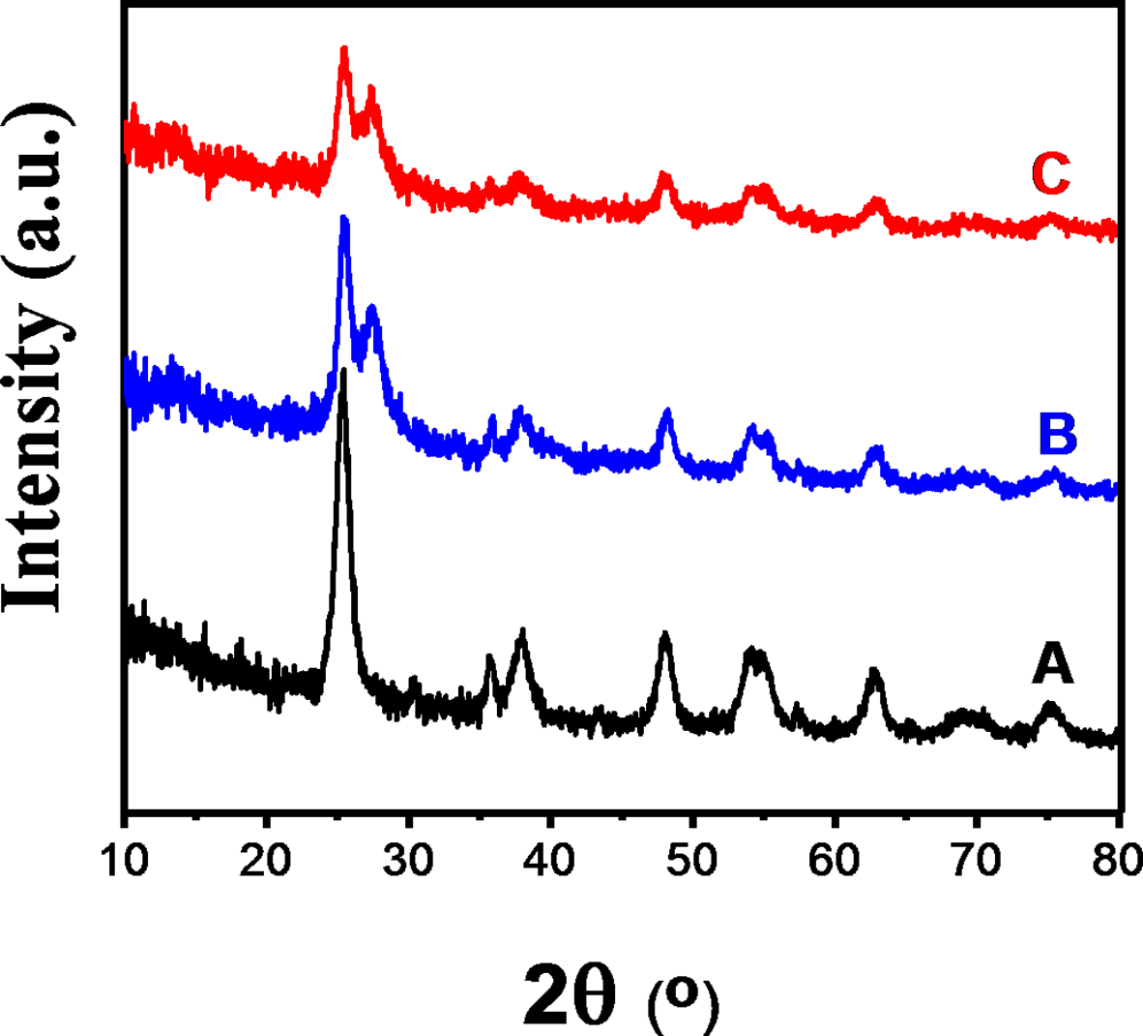
XRD pattern of (**A**) γ-Fe_2_O_3_-TiO_2_, (**B**) g-C_3_N_4_-v@ γ-Fe_2_O_3_-TiO_2_, (**C**) Pd-g-C_3_N_4_-v@ γ-Fe_2_O_3_-TiO_2_ nanohybrid.

Figure 4 depicts the typical XRD patterns of γ-Fe_2_O_3_-TiO_2_, g-C_3_N_4_-v@γ-Fe_2_O_3_-TiO_2,_ and Pd-g-C_3_N_4_-v@γ-Fe_2_O_3_-TiO_2_ heterostructures. For the γ-Fe_2_O_3_-TiO_2_, diffraction peaks at 25.3, 37.8, 47.8, 53.9, and 55.1 were observed (Fig. 4A), corresponding to the (101), (004), (200), (105) and (211) planes of anatase TiO_2_ (JCPDS no. 21-1272)^58^. Also, the XRD pattern of this sample shows diffraction peaks at 30.3, 35.7, 43.3, 57.3, and 62.7° corresponding to the (220), (311), (400), (511), and (440) planes of γ-Fe_2_O_3_ (JCPDS no. 39-1346). As seen in Fig. 4B, a legible diffraction peak corresponding to the (002) lattice plane is observed at 27.3°, which is attributed to the interlayer stacking structure in g-C_3_N_4_^59^. Peaks of γ-Fe_2_O_3_, TiO_2,_ and g-C_3_N_4_ can be found in the Pd-g-C_3_N_4_-v@γ-Fe_2_O_3_-TiO_2_ composite, indicating that the title nanohybrid was formed successfully. The absence of any reflection related to Pd in the XRD pattern of the final hybrid (Fig. 4C) can be caused by the lack of Pd agglomerates demonstrating the uniform distribution of Pd on the catalyst surface which is in agreement with the Pd map (Fig. 3e). Further, the low Pd loading should be taken into account for such an exhibition.

The thermal stability of the Pd-g-C_3_N_4_-v@γ-Fe_2_O_3_-TiO_2_ composite was measured by the TGA from room temperature to 800 ^o^C at a heating rate of 10 ^o^C min^− 1^ (Figure S1). A 3% weight loss at low temperatures (120–224 ^o^C) is assigned to the desorption of physically adsorbed water molecules. The second weight loss in the range of 224–490 °C caused by the decomposition of organic molecules which can be attributed to the breakdown of the g-C_3_N_4_-vanillin junction. The weight loss in the temperature range of 490–640 °C, is related to the decomposition of g-C_3_N_4_ in this region.

**Fig. 5:**
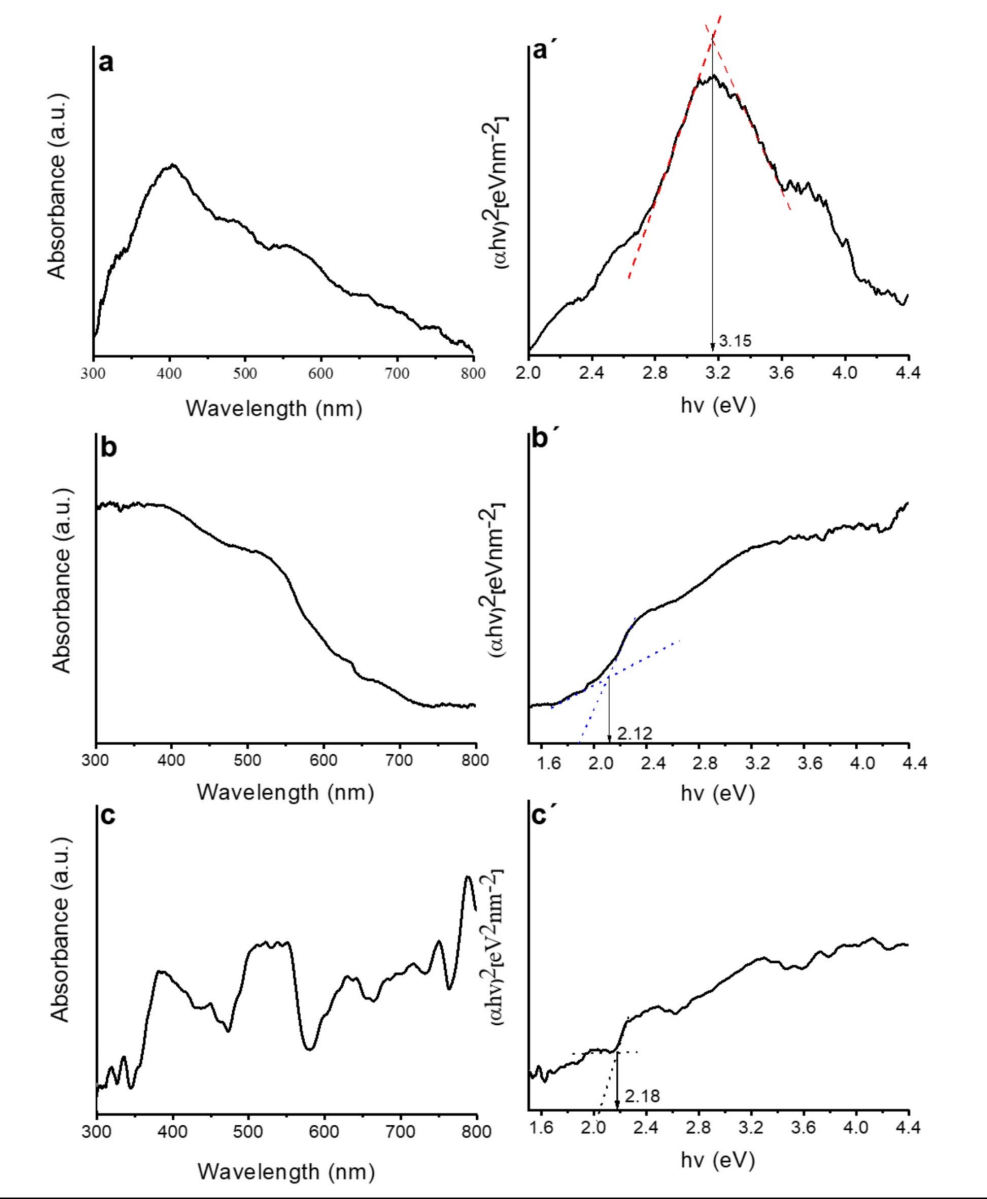
UV-DR spectra and the related Tauc plots of (**a**) γ-Fe_2_O_3_-TiO_2_, (**b**) g-C_3_N_4_-v@ γ-Fe_2_O_3_-TiO_2_, (**c**) Pd-g-C_3_N_4_-v@γ-Fe_2_O_3_-TiO_2_ nanohybrid.

**Fig. 6:**
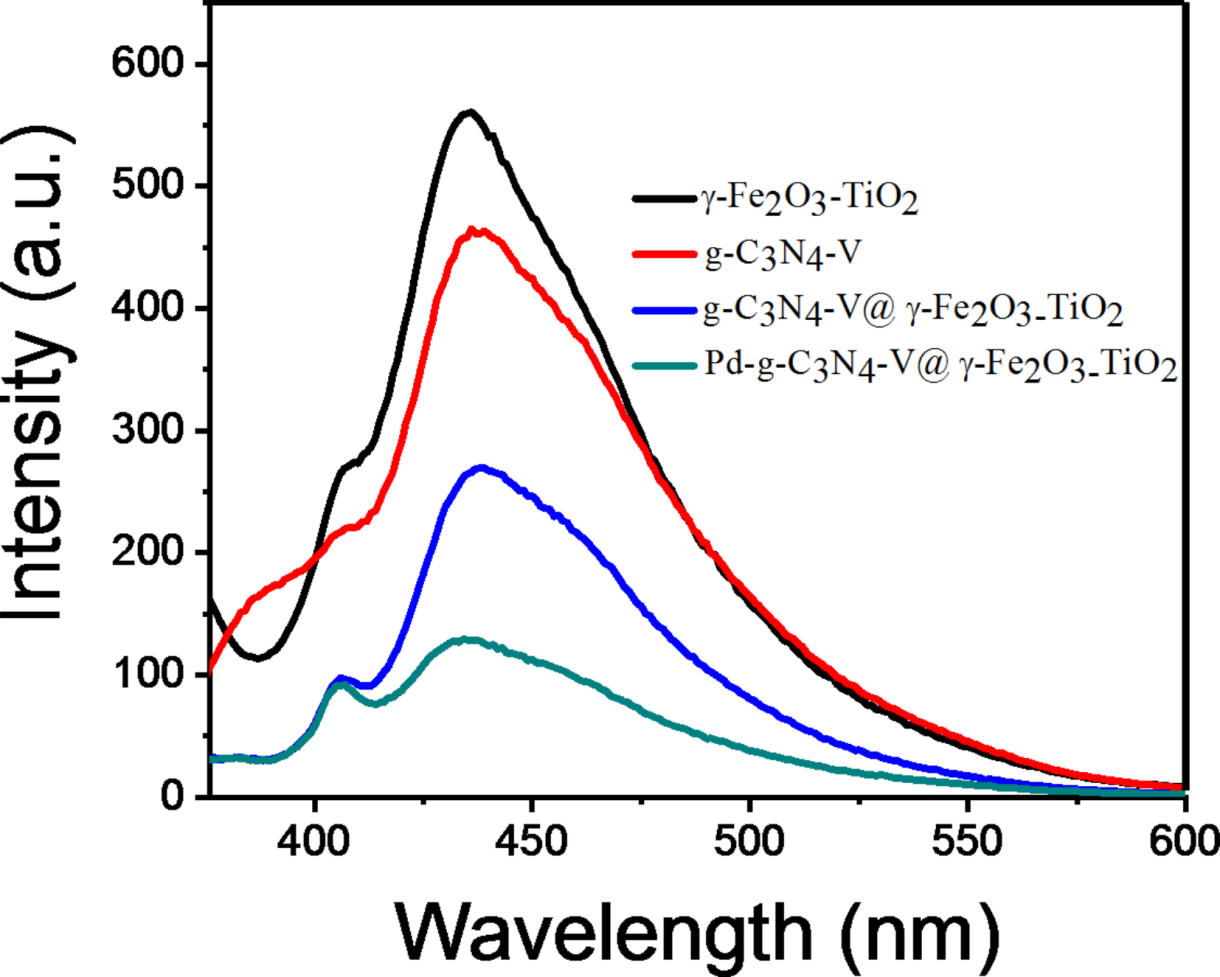
PL analysis of γ-Fe_2_O_3_-TiO_2_, g-C_3_N_4_-v, g-C_3_N_4_-v@γ-Fe_2_O_3_-TiO_2_ and Pd-g-C_3_N_4_-v@ γ-Fe_2_O_3_-TiO_2_ excited at 355 nm.

Diffuse reflectance UV-Vis (DRS) and photoluminescence (PL) spectroscopies were used to assess the optical properties of γ-Fe_2_O_3_-TiO_2_, g-C_3_N_4_-v@ γ-Fe_2_O_3_-TiO_2_, Pd-g-C_3_N_4_-v@ γ-Fe_2_O_3_-TiO_2_ composites with γ-Fe_2_O_3_/TiO_2_ = 1:10 (Figs. 5 and 6). DRS of the materials depicted in Fig. 5a-c showed that the visible light absorption of γ-Fe_2_O_3_-TiO_2_ (Fig. 5a) enhances and widens after functionalization with g-C_3_N_4_-v (Fig. 5b) and particularly Pd loading (Fig. 5c), while its band gap reduced from 3.15 (Fig. 5a´) to 2.12 (Fig. 5b^´^) and 2.18 (Fig. 5c´) respectively. Thus, the Pd-g-C_3_N_4_-v@ γ-Fe_2_O_3_-TiO_2_ nanocomposite possesses a relatively narrow band gap and broader and stronger absorption of visible light expected to be efficient in the visible light region which includes more than 95% of the solar radiation. Nevertheless, the desired photocatalytic activity is achieved if the effective separation of carriers occurs which can be assessed by photoluminescence (PL) spectroscopy. The comparative PL spectra of γ-Fe_2_O_3_-TiO_2_, g-C_3_N_4_-V, g-C_3_N_4_-v@γ-Fe_2_O_3_-TiO_2,_ and Pd-g-C_3_N_4_-v@ γ-Fe_2_O_3_-TiO_2_ excited at 355 nm are given in Fig. 6. The PL intensity of γ-Fe_2_O_3_-TiO_2_ quenched significantly by 52 and 77% after modification with g-C_3_N_4_-v and Pd, respectively, featuring the effective separation of carriers and expected to promote its photocatalytic activity^60^, as will be shown in next sections. The observed changes in the FT-IR spectra of g-C_3_N_4_-v@γ-Fe_2_O_3_-TiO_2_ and Pd-g-C_3_N_4_-v@γ-Fe_2_O_3_-TiO_2_ nanohybrids (Fig. 2c, d) resulted from the formation of new bonds or changing in their strength during the synthesis of materials could explain the observed quenching.

**Fig. 7:**
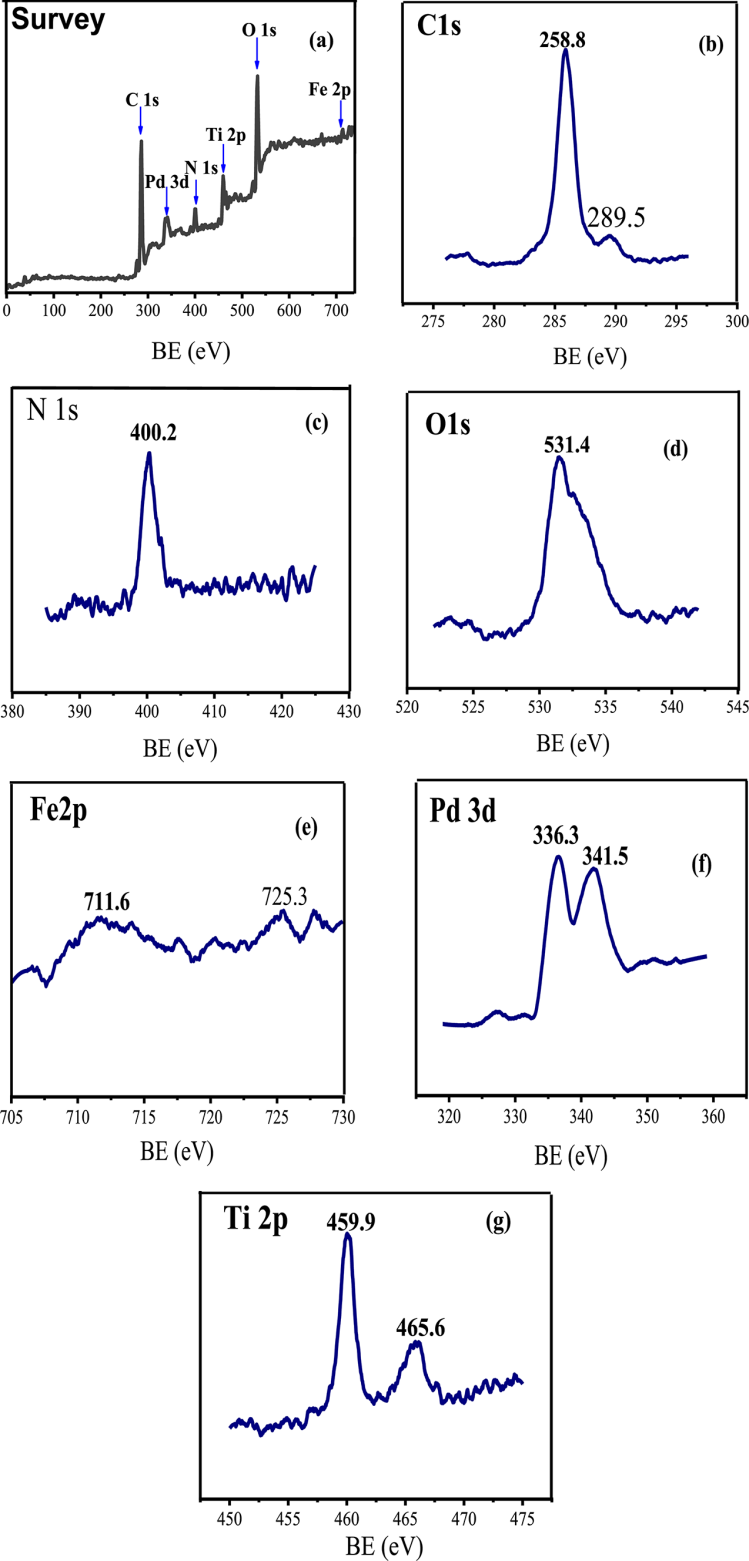
The survey and XPS spectra of the composed elements of Pd- g-C_3_N_4_-v@γ-Fe_2_O_3_-TiO_2_ nanohybrid.

To investigate the surface chemical composition and chemical state of elements in the title Pd catalyst, XPS measurements were carried out in the region from 0 to 1200 eV (Fig. 7). The survey XPS spectrum of Pd- g-C_3_N_4_-v@γ-Fe_2_O_3_-TiO_2_ revealed the presence of C, N, O, Fe, Pd, and Ti elements (Fig. 7a). The predominant peaks at 285.8, 289.5, and 400.2 eV correspond to the C 1s and N 1s of g-C_3_N_4_ (Fig. 7b and c)^61^. The O 1s spectra for the nanocomposites exhibited one distinct peak centered at 531.4 eV, assigned to the O-Fe and O-Ti bonds (Fig. 7d)^62^. The spectrum also showed characteristic low-intensity peaks for Fe in the high-energy regions above 700 eV (Fig. 7e). The observed binding energy for Fe 2p is 725.3 and 711.6 eV, which belong to Fe 2p_1/2_ and Fe 2p_3/2_ of γ-Fe_2_O_3_, respectively^63^. The Pd 3d XPS spectrum showed two peaks at 341.5 and 336.3 eV, corresponding to the Pd 3d_3/2_ and Pd 3d_5/2_ spin − orbit peaks of PdO, respectively (Fig. 7f). A shift to lower binding energy observed for Pd 3d electrons in the as-prepared catalyst compared to the reference values (342.1 and 336.8 eV) indicates that the Pd(II) in the title heterogenized catalyst is more electron-rich than that in pure PdO^64^. Moreover, the peaks of Ti 2p3/2 and Ti 2p1/2 located at 459.9 eV and 465.6 eV, respectively, shifted 1.2 eV and 1.5 eV, to higher binding energy, compared to the reference values of 458.7 and 464.1, respectively, (Fig. 7g), testify the chemical change in the environment resulting from the strong interaction between TiO_2_ with γ-Fe_2_O_3_ NPs^62^.

The magnetic property of Pd-g-C_3_N_4_-v@γ-Fe_2_O_3_-TiO_2_ nanohybrid was investigated by a vibrating sample magnetometer (VSM) at room temperature. Compared with the uncoated γ-Fe_2_O_3_-TiO_2_ nanoparticles, the saturation magnetization of the Pd-g-C_3_N_4_-v@γ-Fe_2_O_3_-TiO_2_ nanohybrid decreased because of the diamagnetic contribution of the organic groups. Despite this reduced magnetization, the solid could still be efficiently separated from the solution with a permanent magnet (Figure S2).

### Catalytic activity

**Fig. 8:**
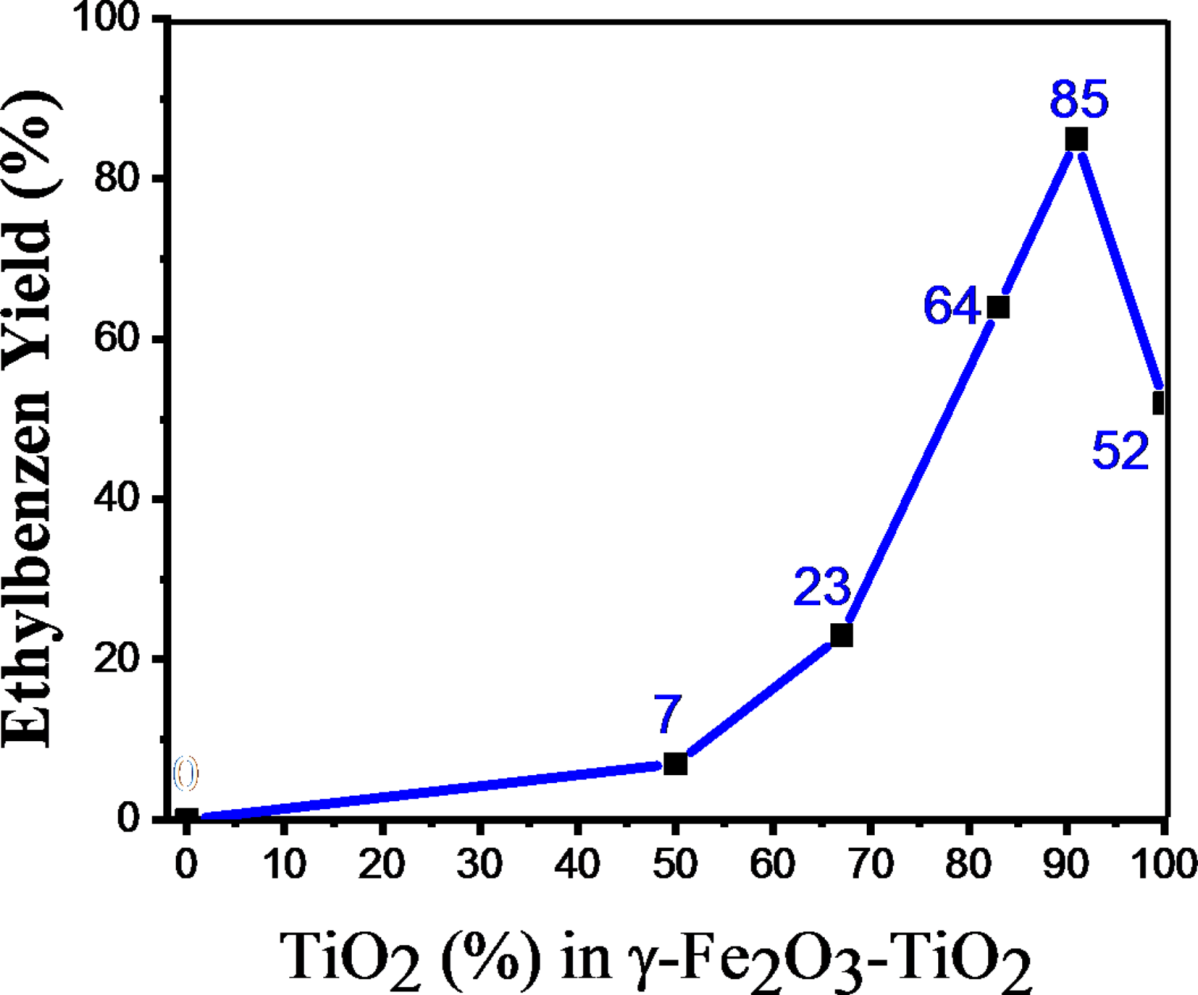
Screening the effect of TiO_2_% on the photocatalytic activity of the Pd-g-C_3_N_4_-v@γ-Fe_2_O_3_-TiO_2_. The yields were determined by GC-FID analysis. Reaction conditions: The reactions were run in ethanol (3 mL) containing 4-chlorobenzaldehyde (0.2 mmol); 1,2- phenylendiamine (0.22 mmol), and styrene (30 µL, 0.26 mmol) at room temperature for 5 h.

 The one-pot reduction of styrene as a model substrate using in situ generated H_2_ at room temperature under visible-light irradiation in the presence of Pd-g-C_3_N_4_-v@γ-Fe_2_O_3_-TiO_2_ composite was chosen for the catalytic activity assessment. The required hydrogen for olefin reduction was supplied by in situ photoassisted acceptorless dehydrogenation of the benzimidazoline intermediate resulting from the condensation of 4-chlorobenzaldehyde (0.2 mmol) and 1,2-phenylenediamine (0.22 mmol) under blue LED^53^. The effect of the weight ratio of γ-Fe_2_O_3_/TiO_2_ in the Pd-g-C_3_N_4_-v@γ-Fe_2_O_3_-TiO_2_ on the photocatalytic performance of styrene hydrogenation was the first factor that screened. For this purpose, the same reaction conditions used in our previous work^53^, were employed. Four different weight ratios of γ-Fe_2_O_3_/TiO_2_ including 1:0, 1:1, 1:2, 1:5, 1:10 and 0:1 corresponding to 0, 50, 67, 83, 91 and 100% TiO_2_ were investigated and the results are summarized in Fig. 8. Inspection of the results shows the significant influence of γ-Fe_2_O_3_/TiO_2_ weight ratios on the photocatalytic evolution of styrene reduction. The hydrogenation performance evolved by increasing the TiO_2_ percentage in the γ-Fe_2_O_3_-TiO_2_ and reached the highest level (85% yield) using 91% TiO_2_ (γ-Fe_2_O_3_/TiO_2_ = 1:10) while the pure TiO_2_-based catalyst (Pd-g-C_3_N_4_-v@TiO_2_) was inferior (52% yield) and γ-Fe_2_O_3_ counterpart (Pd-g-C_3_N_4_-v@γ-Fe_2_O_3_) was dormant. Thus, it appears that the addition of a minor amount (< 10%) of γ-Fe_2_O_3_ to TiO_2_ nanoparticles not only resolves the problem of separation of catalyst from the reaction mixture but also promotes notably its photocatalytic activity towards styrene reduction featuring the synergistic effect between γ-Fe_2_O_3_ and TiO_2_ in the photocatalytic process.

With the best γ-Fe_2_O_3_/TiO_2_ ratio in hand, screening of other factors affecting the photocatalytic performance of Pd-g-C_3_N_4_-v@γ-Fe_2_O_3_-TiO_2_ was put on the agenda. The results of the solvent effects, catalyst amount, and reaction atmosphere on the synthesis of benzimidazole using 4-chlorobenzaldehyde (0.2 mmol) and 1,2-phenylenediamine (0.22 mmol) as the source of hydrogen generation in the reaction vessel are given in the Figure S3. Several solvents such as EtOH, n-Hexane, H_2_O, DCM, MeOH, EtOAc, CH_3_CN (0.5 mL), as well as solvent-free conditions in the presence of 3 mg of catalyst, were investigated. EtOH as a green solvent was the best because the yield of benzimidazole was significantly higher than the others (Figure S3A). Various amounts of the Pd-g-C_3_N_4_-v@γ-Fe_2_O_3_-TiO_2_ catalyst and ethanol were also examined and the best performance was achieved using 2 mg of catalyst and 3 mL ethanol. (Fig S3B, C).

### Photocatalytic reduction of olefins

**Fig. 9:**
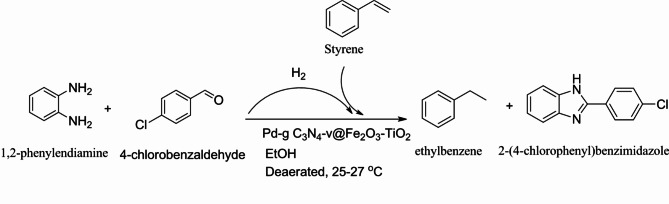
Photocatalytic generation of hydrogen gas from the catalytic condensation of 1,2-phenylendiamine and 4-chlorobenzaldehyde followed by Styrene hydrogenation.

**Fig. 10:**
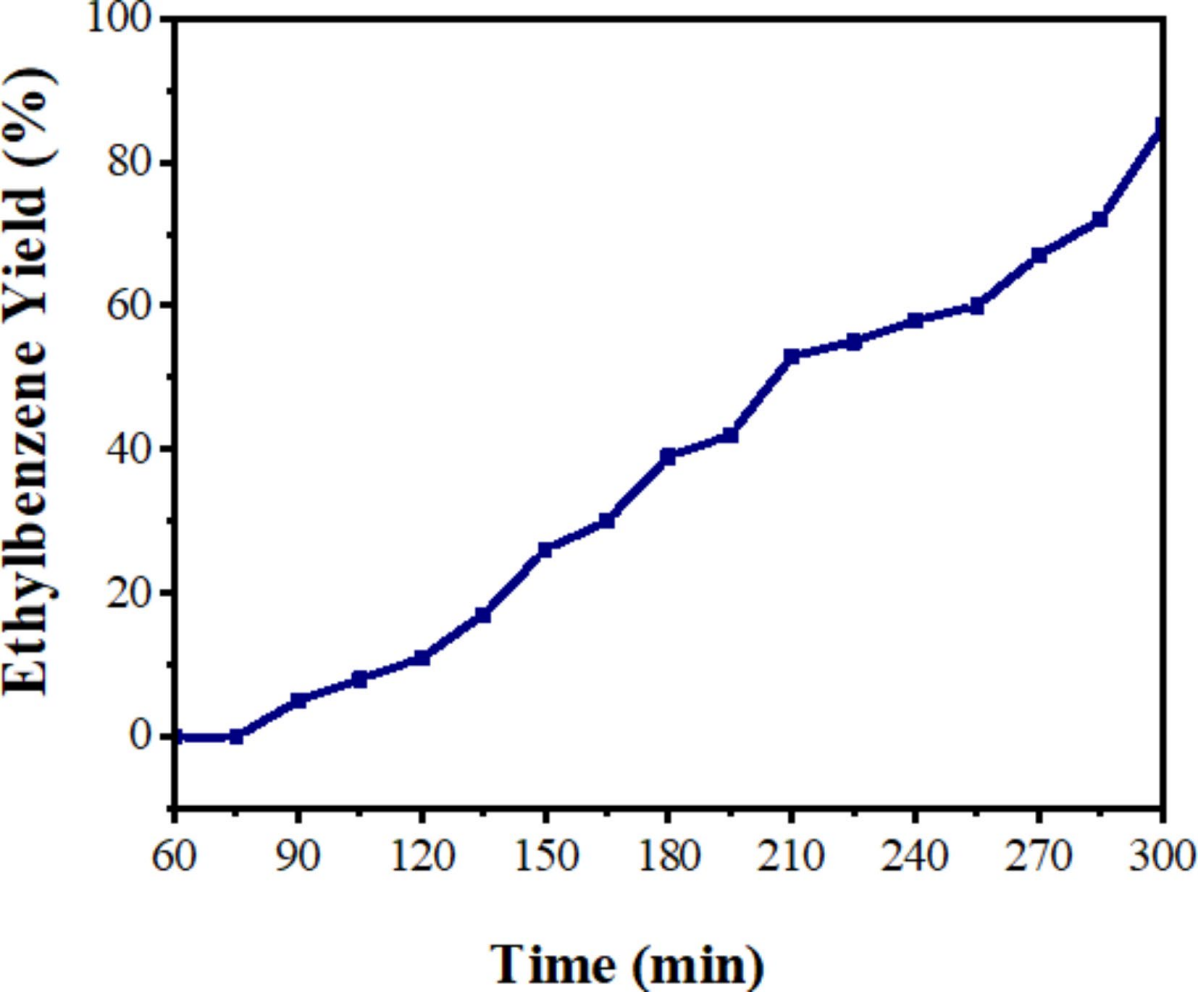
Time course of photoassisted production of ethylbenzene using in situ generated H_2_ in the presence of the Pd-g-C_3_N_4_-v@ γ-Fe_2_O_3_-TiO_2_ nanohybrid under blue LED irradiation.

 Knowing that the hydrogen gas is produced from the dehydrogenation of benzimidazoline intermediate during the synthesis of benzimidazole^53^, one pot photoreduction of olefins to the saturated hydrocarbons by in situ generated hydrogen was carried out (Fig. 9). For this, the catalyst (Pd-g-C_3_N_4_-v@γ-Fe_2_O_3_-TiO_2_) was added to a test tube containing 4-chlorobenzaldehyde and 1, 2-phenylenediamine dissolved in EtOH. The tube was sealed with a septum cap, and the reaction mixture was deaerated for 5 min (Ar). Then, 30 µL of olefin was injected into the reaction mixture and the tube was transferred into a reactor chamber equipped with a magnetic stirrer. The mixture was stirred at room temperature for 5 h under a blue light-emitting diode (LED, 40 W) with an intensity of 1.1 W/cm^2^. The yield of the produced ethylbenzene from the hydrogenation of styrene was monitored by GC-FID at 15 min intervals (Fig. 10) to evaluate the catalytic activity of Pd-g-C_3_N_4_-v@ γ-Fe_2_O_3_-TiO_2_.

To confirm the superiority of the title photocatalyst, the catalytic activity of the parent and precursor materials was assessed (Figure S4). Bare TiO_2_, g-C_3_N_4_, γ-Fe_2_O_3_-TiO_2_, g-C_3_N_4_-v, Pd-C_3_N_4_-v, g-C_3_N_4_-v@γ-Fe_2_O_3−_TiO_2_, Pd-g-C_3_N_4_-v@γ-Fe_2_O_3_, Pd-g-C_3_N_4_-v@TiO_2_ as well as Pd(OAC)_2_, and even the mixture of Pd(OAc)_2_ and g-C_3_N_4_-v@γ-Fe_2_O_3_-TiO_2_ were inferior or quite ineffectiveness. It should be noted that when a different protocol (Figure S5) was used for the preparation of magnetically recyclable photocatalyst as Pd-g-C_3_N_4_-v-g-Fe_2_O_3_@TiO_2_, the conversion of styrene to ethylbenzene under optimized conditions reduced to 60%.

These results demonstrated well the critical role of Pd NPs stabilized on g-C_3_N_4_-v imine grafted on γ-Fe_2_O_3_-TiO_2_ mixed oxides NPs, and highlighted the synergistic effects of the catalyst precursors in the constructed photocatalyst for enhancing the photocatalytic activity as depicted in a proposed mechanism in Figure S6 (SI).

With optimized conditions in hand, the scope of the method was extended to a variety of olefins, and the styrenes substituted with Cl, and CH_3_ (Table 1, entries 2–4) showed high activity towards visible light-assisted hydrogenation at room temperature and the pertinent ethylbenzenes were produced in high yields. Further, the benzimidazole product (Table 1, product A) was produced absolutely and generation of the product B ceased (Table 1) rendering a zero-waste catalytic reaction. Allyl alcohol (Table 1, entry 5), and allyl ketone (Table 1, entries 6) conjugated to the phenyl ring were also reduced successfully in good yields. However, 1-cyclohexen-2-one reduced moderately (44%) and non-conjugated allylbenzene (Table 1, entry 8) was dormant under different conditions. The chemoselectivity of the hydrogenation reaction was also notable. While, the olefin moiety was successfully hydrogenated, the aromatic and carbonyl groups remained intact. Looking for an advantage of the present photocatalytic system over our previous work, we found a higher yield of the hydrogenated α, β-unsaturated ketones (Table 1, entries 6 and 7) and higher selectivity for benzimidazole formation (product A) testifies to higher atom economy of the present catalytic system.

### Photocatalytic assessment

**Fig. 11:**
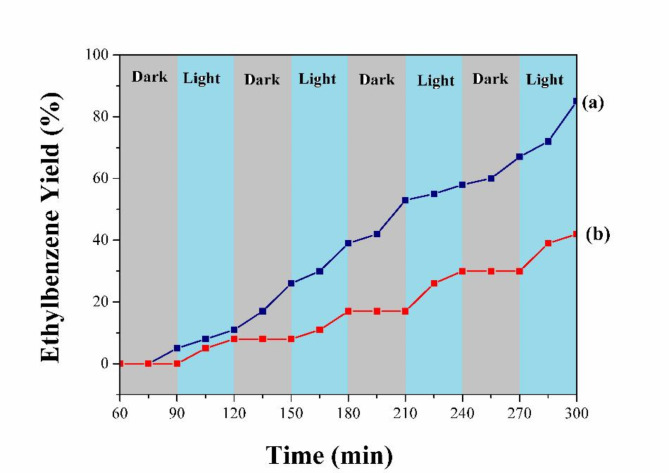
Comparison between light-dark cycles (red) and continuous irradiation (blue) under the blue LED light. The yields are determined by GC-FID analysis.

 The photocatalysis nature of the reaction was initially explored by the light − dark cycle in the hydrogenation of styrene. The production of ethylbenzene ceased in the dark and was restored upon blue LED light irradiation (Fig. 11). Then, the dependence of the catalytic activity on the light source was assessed. Based on the results summarized in Table 2, the catalytic performance is affected significantly by the light source and intensity. The blue 40 w LED was the best light source in terms of conversion rate and selectivity of the benzimidazole product (A in Table 2) enhancing significantly the atom economy of the reaction system. Nevertheless, the 12 W blue LED and other lights reduced the reaction rate and mainly diverted the reaction pathway to the over-condensation product (product B in Table 2).

**Table 1:** Photocatalytic Reduction of Olefins from condensation of 1,2 phenylenediamines and 4-Chlorobenzaldehyde in the Presence of Pd-g-C3N4-V@-Fe2O3-TiO2a.


Entry	R	Hydrogenated Olefin % ^b^	Product A %	Product B %
1	H	85	100	0
2	4-Cl	95	100	0
3	2-CH_3_	82	100	0
4	4-CH_3_	90	100	0
5		63	75	25
6		78	100	0
7		44	60	40
8		0	45	55

^a^ The reactions containing 4-chlorobenzaldehyde (0.2 mmol); 1,2-phenylendiamine (0.22 mmol), and substituted styrenes (30 µL, 0.26 mmol) were run in ethanol (3 mL) containing 2 mg of Pd-g-C_3_N_4_-v@γ-Fe_2_O_3_-TiO_2_ at room temperature for 5 h. ^b^ GC yield based on chlorobenzene as an internal standard.

**Table 2:** Experiments for photocatalytic styrene reduction in different lamps a.


Entry	Lamp	Hydrogenated Olefin % ^b^	Product A %	Product B %
1	Blue LED 40 w	85	100	0
2	CFL	74	100	0
3	UV Light	18	30	70
4	Blue LED (12 w)	20	50	50
5	Actinic BL	0	20	80
6	Reptillight	0	5	95
7	Dark	0	20	40

^a^ The reactions containing 4-chlorobenzaldehyde (0.2 mmol);1,2-phenylenediamine (0.22 mmol), and styrene (30 µL, 0.26 mmol) were run in ethanol (3 mL) containing 2 mg of Pd-g-C_3_N_4_-v@γ-Fe_2_O_3_-TiO_2_ at room temperature for 5 h. ^b^ GC yield.

The action spectrum also revealed that the reaction is wavelength dependent. For this, the rates of the styrene reduction associated with the formation of 2-[4-chlorophenyl] benzimidazole in the presence of Pd-g-C_3_N_4_-v@γ-Fe_2_O_3_-TiO_2_ under a full spectrum CFL bulb using different cut-off filters were determined. As shown in Figure S7, a good correlation is observed between the apparent quantum yield (AQY) and DRS of the photocatalyst which confirms that the reaction is taking place photocatalytically.

### Stability analysis of Pd-g-C_3_**N**_4_-v @γ-Fe_2_O_3_-TiO_2_nanohybrid

 One of the most important parameters related to magnetic materials is their stability and reusability. At the end of the reaction, the catalyst was isolated by an external magnet and washed with ethanol (3 × 5 mL ethanol), followed by drying in a vacuum oven to be reused for the next runs. The photocatalytic stability and recyclability of the Pd-g-C_3_N_4_-v@γ-Fe_2_O_3_-TiO_2_ nanohybrid were analyzed by five successive cyclic photohydrogenation of styrene experiments and shown in Figure S8.

It was found that the Pd-g-C_3_N_4_-v@γ-Fe_2_O_3_-TiO_2_ nanohybrid maintained its catalytic activity under photochemical conditions and almost the same product yield was observed for ethylbenzene after each run even the fifth run. Further, the amount of palladium leaching was also determined in the fresh and the reused catalyst by ICP-OES analysis (0.10 mmolg^− 1^), and no leaching of palladium was observed. FT-IR spectra after 5 cycles of photohydrogenation of styrene demonstrated that the photocatalyst structure remains unchanged. (Figure S8B). These results confirmed that the γ-Fe_2_O_3_-TiO_2_, g-C_3_N_4_-v imine, and Pd nanoparticles are well connected strongly.

### Comparison study

 Finally, we tried to compare our results with previously reported works while believing that making such a comparison is difficult. The in situ H_2_ generation from an acceptorless dehydrogenation of benzimidazoline intermediate followed by the hydrogenation of olefins under visible-light and mild reaction conditions to produce both benzimidazoles and saturated hydrocarbons in high yields and excellent selectivity and in a single pot is an unprecedented activity as far as we know^53^. However, the hydrogen generation and reduction of olefin using catalysts based on Pd, TiO_2_, or g-C_3_N_4_ in the literature, may be a criteria for comparison. As summarized in Table S1, most of the reported catalysts are unable to perform both processes in a single pot and require a reducing agent or external hydrogen source for olefin reduction. Some of the reported methods have serious limitations for substrate diversity and are limited to one olefin substrate. Some of them were time-consuming and used hazardous solvents and reagents, harsh and tedious work-up procedures, as well as UV-light or high-power Xe lamps (500–1000 W), which are further drawbacks that cannot be ignored for industrial implementations.

## Conclusion

In summary, we developed an effective visible-light-assisted dehydrogenation/hydrogenation catalytic system using Pd-g-C_3_N_4_-v@γ-Fe_2_O_3_-TiO_2_ as a magnetically separable catalyst. Vanillin as a natural junction that efficiently binds Pd/g-C_3_N_4_ to γ-Fe_2_O_3_-TiO_2_ mixed oxide nanoparticles via an imine bond produces a highly active, air-stable, robust, magnetically recyclable photocatalyst. Synchronous H_2_ generation, from an acceptorless dehydrogenation of benzimidazoline intermediate, and hydrogenation of olefins were successfully achieved with no/low waste production in the presence of the fabricated catalyst under a blue LED light and mild conditions. The right weight ratio of γ-Fe_2_O_3_:TiO_2_ (1:10) in nanocatalyst plays a critical role in promoting the photocatalytic performance of Pd-g-C_3_N_4_-Vanillin@γ-Fe_2_O_3_-TiO_2_ toward olefins hydrogenation. The effective charge separation of the photocatalyst promoting its photoefficiency was provided by modification with Pd loaded g-CN_4_-Vanillin evidenced by PL spectra. The light-dependence photocatalysis and effective visible-light responsivity of the photocatalyst were approved by action spectra. Compared with the previous reports, this protoc_3_ol provides a direct approach for the preparation of saturated hydrocarbons from olefins under very mild reaction conditions without any additives or external hydrogen gas sources. Reducing/removing the waste, and performing the reaction under heterogeneous conditions in ethanol as a green solvent under perfectly safe light sources are additional benefits of our work testifying that the present work is atom efficient and matches with the green chemistry principles. In addition, the replacement of TiO_2_ NPs in our previous work, with a magnetically separable mixed metal oxide counterpart (γ-Fe_2_O_3_-TiO_2_ NPs) as well as commercially unavailable and unsafe (3-oxopropyl)trimethoxysilan with vanillin as a natural and safe product to join the g-C_3_N_4_ to γ-Fe_2_O_3_-TiO_2_ through an easy condensation of g-C_3_N_4_ with vanillin are other important advantages of the present catalyst than that of our previous one. This strategy creates new opportunities to fabricate natural-based multi-purpose catalysts for synchronous eco-friendly processes.

## Experimental

General remarks and multi-step synthesis procedures of Pd-g-C_3_N_4_-vanillin@γ-Fe_2_O_3_-TiO_2_ catalyst are given in supporting information.

### General procedure for the photocatalytic reduction of Olefins

 In a typical experiment, 2 mg of Pd- g-C_3_N_4_-v@γ-Fe_2_O_3_-TiO_2_ nanocatalyst was added to a test tube containing 4-chlorobenzaldehyde (0.2 mmol) and 1, 2-phenylenediamine (0.22 mmol) dissolved in EtOH (3 mL). The tube was sealed with a septum cap, and the reaction mixture was deaerated for 5 min (Ar). 30 µL (0.26 mmol) of olefin was injected into the reaction mixture and the tube was transferred into a reactor chamber equipped with a magnetic stirrer. The mixture was stirred at room temperature for 5 h under a blue light-emitting diode (LED) (40 W). At the end of the reaction, the solid catalyst was removed by an external magnet and the yield and characterization of the products were determined by gas chromatography (GC-FID) analysis using chlorobenzene as an internal standard.

## Electronic supplementary material

Below is the link to the electronic supplementary material.

Supplementary Material 1

## Data Availability

Data is provided within the manuscript or supplementary information files.
