# Supplementary material for: Synchronous photocatalytic benzimidazole formation and olefin reduction by magnetic separable visible-light-driven Pd-g-C3N4-Vanillin@γ-Fe2O3-TiO2 Nanocomposite
Source: Sci Rep. 2024 Nov 6;14:27017. doi: 10.1038/s41598-024-76198-z (PMC11541593; doi:10.1038/s41598-024-76198-z)
Supplement: Supplementary file 1 — Supplementary Material 1 [file 41598_2024_76198_MOESM1_ESM.docx]

**Supporting Information**

**Synchronous** [**Photocatalytic**](https://pubs.acs.org/doi/abs/10.1021/acs.inorgchem.3c00250) **Benzimidazole Formation and Olefin Reduction by Magnetic Separable Visible-Light-Driven** **Pd-g-C_3_N_4_-Vanillin@γ-Fe_2_O_3_-TiO_2_ Nanocomposite**

Maasoumeh Jafarpour, ^a, c*^ Abdolreza Rezaeifard, ^b, c *^ Narges Pourmorteza, ^c,^ ^d^ Maryam GhanbariKudeyani ^c^

^a^ Department of Organic Chemistry, Faculty of Chemistry and Petroleum Sciences, Bu-Ali Sina University, Hamedan, 65178-38695, Iran. Email: [m.jafarpour@basu.ac.ir](mailto:%20m.jafarpour@basu.ac.ir)

**^b^** Department of Inorganic Chemistry, Faculty of Chemistry and Petroleum Sciences, Bu-Ali Sina University, Hamedan, 65178-38695, Iran. Email: a.rezaeifard@basu.ac.ir

^c^ Catalysis Research Laboratory, Department of Chemistry, Faculty of Science, University of Birjand, Birjand, 97179-414 Iran.

**^d^** South Raadab Engineering Company, Zahedan Desalination Plant, 98169-13419 Iran.

**Experimental**

**Instrumentation.** FT-IR spectra were recorded on a JASCO 6300 spectrometer in the range of 400-4000 cm^-1^. Thermogravimetric analysis (TGA) of powders carried out on NETZSCH TG 209 F1 Iris under N_2_ atmosphere flow at a uniform heating rate of 10 ˚Cmin^-1^ in the range of 30-890 ˚C. The Pd content of the catalyst was measured by an inductively coupled plasma optical emission spectroscopy (ICP-OES), using a VISTA-PRO ICP analyzer. Diffuse reflectance UV-vis spectra were recorded using an Avaspec Avantes spectrometer (Avaspec-2048-TEC model). EDX spectrum was recorded by the TESCAN Vega Model instrument. X-ray photoelectron spectroscopy measurements were recorded using a BESTEC GMBH (10^-10^ mw) with Al anode. The Field Emission Scanning Electron Microscopy (FE–SEM) images were taken on a Tescan MIRA3. Powder X-ray diffraction (XRD) was performed on a Bruker D8-advance X-ray diffractometer with Cu Kα (λ=1.54178 Å) radiation. Room temperature magnetization isotherms were obtained using a vibrating sample magnetometer (VSM, LakeShore 7400). Photoluminescence (PL) measurements were recorded on the RF-5301DCM SHIMADZU instrument. Progresses of the reactions were monitored by TLC using silica-gel SIL G/UV 254 Plates. The yield of the hydrogenated products was determined by GC-FID on a Shimadzu GC-16A instrument using a USA RESTEK 30 m Rtx^@^-1 column (0.25 mm ID and 0.25 μm). The light sources are Reptile lamp, LT NARVA (18 W, full range visible light + 4% UV), Actinic BL TL-D Philips (15 W, *λ* = 366−400 nm), and blue LED, AC86, Z.F.R (12 W, *λ*max = 505 nm), UV light (λ =200-290 nm, 15 W), Blue LED (Kessil A160WE, 40 W, 390-500 nm) and room light lamps (Fluorescent lamp, λ = 400-800 nm, P= 40 W).

**Preparation of γ-Fe_2_O_3_.** γ-Fe_2_O_3_ nanoparticles were prepared by a reported chemical co-precipitation technique of ferric and ferrous ions in an alkali solution with minor modifications ^1^. FeCl_2_. 4H_2_O (1.99 g, 10 mmol) and FeCl_3_. 6 H_2_O (3.25 g, 12 mmol) were dissolved in deionized water (30 mL) under an Ar atmosphere at room temperature. An ammonia solution (NH_4_OH, 0.6 M, 200 mL) was added dropwise (drop rate =1 mL min^-1^) to the stirring mixture at room temperature to reach the reaction pH of 11. The resulting black dispersion was continuously stirred for 1 h at room temperature and then heated to reflux for 1 h to yield a brown dispersion. The magnetic nanoparticles were then separated by an external magnet and washed with deionized water until it was neutralized. The as-synthesized sample was heated at 2 °C min^-1^ up to 250 °C and then kept in the furnace for 3 h to give a reddish-brown powder.

**Preparation of γ-Fe_2_O_3_-TiO_2_ nanoparticle.** γ-Fe_2_O_3_-TiO_2_ nanoparticles with different TiO_2_ content were fabricated by hydrothermal method.^2^ Synthesis of all ratios of γ-Fe_2_O_3_-TiO_2_ nanoparticle was carried out using 0.1 g γ-Fe_2_O_3_ but the feeding amount of tetrabutylorthotitanate (TBOT) was varied in order to obtain composites with varying TiO_2_ to γ-Fe_2_O_3_ ratio. Initially, 0.1 g of γ-Fe_2_O_3_ is dispersed in 5 ml of deionized water through sonication for 30 min. Then, tetrabutylorthotitanate (TBOT) and citric acid (CA) with a molar ratio of 1:3 was added to the deionized water (5 mL) and the resulting solution was dropped into the γ-Fe_2_O_3_ suspension under sonication at room temperature. The 1:3 molar ratio of TBOT and CA was maintained for all the synthesis experiments.

The amount of TBOT for different samples was maintained at 0.1, 0.2, 0.5, and 1 g to study the effects of chemical proportions on the photochemical properties of nanohybrid. Then, each mixture is transferred into a 47 mL Teflon-lined stainless steel autoclave and heated at 180 ^o^C for 10 h. After the hydrothermal reaction, the autoclave was cooled to room temperature and the precipitates were washed with deionized water and ethanol several times and then dried at 60 ^o^C for 2 h in a vacuum oven. Finally, γ-Fe_2_O_3_-TiO_2_ composites were achieved by calcination of the as-collected composite nanostructures at 450 ^o^C for 2 h.

**Preparation of g-C_3_N_4_ powder.** Graphite-like C_3_N_4_ sheets were prepared based on a procedure reported previously. Specifically, 5.0 g of melamine was calcined at 550 °C for 4 h with a heating rate of 2 °C/min in a muffle furnace covered with a lid to maintain the calcination process in static air. The obtained yellow agglomerates were collected and ground into powder for further synthesis.^3^

**Preparation of g-C_3_N_4_-vanillin Schiff base (g-C_3_N_4_-v).** For the synthesis of g-C_3_N_4_-Imine, a mixture of 0.1 g of g-C_3_N_4_ and 2.5 mmol of vanillin was ground thoroughly. Then the mixture was put in an alumina crucible with a cover and heated at 100-W microwave power.

**Preparation of Pd-g-C_3_N_4_-v@γ-Fe_2_O_3_ -TiO_2_ nanohybrid.** First, 0.1 g of γ-Fe_2_O_3_ -TiO_2_ nanoparticles was gradually added to 0.1 g of g-C_3_N_4_-v Schiff base in ethanol at 60 ^o^C under ultrasonic agitation. The as-obtained mixture was refluxed for 8 h. Then, the product was centrifuged and separated with a permanent magnet, and washed with ethanol. Finally, g-C_3_N_4_-v@γ-Fe_2_O_3_@TiO_2_ nanocomposite was obtained after drying in air. Subsequently, to load Pd nanoparticles on the surface of the g-C_3_N_4_-v@γ-Fe_2_O_3_-TiO_2_ heterostructure, a volume of Pd(OAc)_2_ suspension (4.2 mg per mL ethanol) was added to 0.1 g of g-C_3_N_4_-v@γ-Fe_2_O_3_-TiO_2_ dispersed in 2 mL ethanol. After 1 h sonication, the mixture was refluxed for a further 3 h. The black precipitates were collected by centrifuging and separated with a permanent magnet. Then, the product was washed with ethanol repeatedly and dried in the air.

**Fig. S1.** TGA curve of Pd-g-C_3_N_4_-v@ γ-Fe_2_O_3_-TiO_2_ nanohybrid.

**Fig. S2**. Magnetization curve of (a) ­γ-Fe_2_O_3_-TiO_2_ (b) g-C_3_N_4_-v@ γ-Fe_2_O_3_-TiO_2_ nanohybrid and (c) Pd- g-C_3_N_4_-v@ ­γ-Fe_2_O_3_-TiO_2_ nanohybrid.

**Fig. S3**. The screening of the solvent nature (3 mL) (a), amount of EtOH as solvent (b), and catalyst dosage (c) in the synthesis of benzimidazole using 4-chlorobenzaldehyde (0. 2 mmol), 1,2-phenylendiamine (0.22 mmol) under blue LED irradiation.

**Figure S4.** Comparison of the photocatalytic activity of the parent and precursor materials with Pd-g-C_3_N_4_-v@ γ-Fe_2_O_3_-TiO_2_ in the photocatalytic styrene reduction. The reactions containing 4-chlorobenzaldehyde (0.2 mmol); 1,2-phenylendiamine (0.22 mmol), and styrene (30 μL, 0.26 mmol) were run in ethanol (3 mL) at room temperature for 5 h under a Blue LED (40 W) with the intensity of 1.1 W/cm^2^. The yields were determined by GC analysis.

**Figure S5.** Synthetic route of magnetically recyclable photocatalyst of Pd- g-C_3_N_4_-v- γ-Fe_2_O_3_@ TiO_2_ nanocatalyst

**Figure S6.** The proposed photocatalytic mechanism for one-pot olefin reduction using in situ photogenerated H_2_ during benzimidazole formation over Pd-g-C_3_N_4_-Vanillin@γ-Fe_2_O_3_-TiO_2_ under visible light irradiation.

**Fig. S7**. Photocatalytic action spectrum for reduction of styrene associated with synthesis of 2-[4 chlorophenyl] benzimidazole using Pd-g-C_3_N_4_-v@γ-Fe_2_O_3_-TiO_2_ photocatalyst under a full spectrum CFL bulb (400-800 nm)

**Fig. S8. (A)** Recycling of the catalytic system for the photohydrogenation of styrene in the presence of Pd-g-C_3_N_4_-v@γ-Fe_2_O_3_-TiO_2_ nanohybrid. (B) Comparison of FTIR spectra of fresh Pd-g-C_3_N_4_-v@γ-Fe_2_O_3_-TiO_2_ (a) with a used one (b) in Photocatalytic Reduction of Olefins after 5 runs.

| **Table S1**. Comparison of various catalytic methods based on Pd, TiO_2_ or g-C_3_N_4_ for generation of hydrogen and reduction of styrene. | | | | | | |
| --- | --- | --- | --- | --- | --- | --- |
| Entry | Catalyst | Conditions | H_2_ production | Olefin reduction | Conv. %^a^ | Ref. |
|  | Pd-g-C_3_N_4_-Vanillin@γ-Fe_2_O_3_-TiO2 | Blue LED, r.t., 5 h, Ethanol  (one pot) | **+** | **+** | 85 | This work ^b^ |
| 1 | Pd- g-C_3_N_4_ Imine/TiO_2_ | Blue LED, r.t., 5 h, Ethanol  (one pot) | **+** | **+** | 78 | ^3^ |
| 2 | Pd/MOF@COF | Visible ligh, EtOH, NH_3_BH_3_, 1 min (dual-chamber microreactor) | **+** | **+** | 94 | ^4^ |
| 3 | Pd/g-C_3_N_4_ | Water, NH_3_BH_3_, 40°C, 18 h (emulsion microreactors) | **+** | **+** | 97 | ^5^ |
| 4 | Pd-g-C_3_N_4_ | LED 15 W, r.t, 12 h, NiCl_2_ | **_** | **+** | 95 | ^6^ |
| 5 | Pd@NPCc | EtOH, HCO_2_H, NH_4_CO_2_H, 1h | **_** | **+** | 98 | ^7^ |
| 6 | NiPt/C_3_N_4_ | N_2_H_4_, NaOH, 50°C | **+** | **_** | _ | ^8^ |
| 7 | Pd/TiO_2_ | H_2_, r.t, | **_** | **+** | >99 | ^9^ |
| 8 | Pd/TiO_2_ | EtOH, 30°C , H_2_, 1 h | **_** | **+** | 100 | ^10^ |
| 9 | Fe@g-C_3_N_4_ | 40 W domestic light bulb, NH_2_NH_2_, H_2_O, 8h | **_** | **+** | 98 | ^11^ |
| 10 | Pd/CeO_2_ | 60°C , H_2_, 4 h | **_** | **+** | 70 | ^12^ |
| 11 | Pd(OAc)_2_ | Xantphos, water, 90°C , 20 h | **_** | **+** | 99 | ^13^ |
| 12 | MWCT/Pd@TiO_2_ | UV light or simulated, EtOH | **+** | **_** | _ | ^14^ |
| 13 | Pd-TiO_2_ | UV light, 2-propanol, 30 min | **_** | **+** | >99 | ^15^ |
| 14 | Pd, Pd-TiO_2_ | Visible light, water, r.t, H_2_ | **_** | **+** | 60 | ^16^ |
| 15 | Pd-mpgd-C_3_N_4_ | HCOOH, EtOH, r.t | **_** | **+** | 58 | ^17^ |
| 16 | g-C_3_N_4_ | 300 W Xe lamp, TEOA, Pt | **+** | **_** | _ | ^18^ |
| 17 | g-C_3_N_4_/NiS | 300 W Xe lamp, TEOA | **+** | **_** | _ | ^19^ |
| 18 | Pd/Ga(OH)_3_ | EtOH, 25°C, H_2_, 1.3 h | **_** | **+** | 100 | ^20^ |
| 19 | Pd_UV-H2_/Sm_2_O_3_ | EtOH, 35°C, H_2_, 90 min | **_** | **+** | 100 | ^21^ |
| 20 | Pd/TiO_2_/g-C_3_N_4_ | 300 W Xe lamp, H_2_O:EtOH | **+** | **_** | **_** | ^22^ |
| 21 | Pd–Rb_2_O@g­C_3_N_4_/TiO_2_ | Solar irradiation, PH= 8 | **+** | **_** | **_** | ^23^ |
| 22 | TiO_2_/CdS/ g-C_3_N_4_ | 1000 W Xe lamp, Na_2_S/ Na_2_SO_3_ | **+** | **_** | **_** | ^24^ |
| 23 | Pd/N-HTC^e^ (n.r.) | HCOONa, 90°C | **+** | **_** | **_** | ^25^ |
| 24 | Pt-Pd/ Ni/ C | EtOH, - 25°C, H_2_, 3.5 h | **_** | **+** | 100 | ^26^ |
| 25 | Pd@M^f^-C16 | H_2_O, H_2_, 60 min | **_** | **+** | 100 | ^27^ |
| ^a^ Ethylbenzene conversion. ^b^ 4-chlorobenzaldehyde (0.2 mmol); 1,2-phenylendiamine (0.22 mmol); styrene (30 µL) at room temperature in ethanol (3 mL). ^c^ Nitrogen-enriched porous carbon nanosheets. ^d^ Mesoporous carbon nitride. ^e^ The carbon support consisted of a H_3_PO_4_-assisted hydrothermal carbonization. ^f^ micro-organohydrogels. | | | | | | |

**References**

(1) Rezapour, E., Jafarpour, M., & Rezaeifard, A. Palladium niacin complex immobilized on starch-coated maghemite nanoparticles as an efficient homo-and cross-coupling catalyst for the synthesis of symmetrical and unsymmetrical biaryls. *Catalysis Letters*, **2018**, *148*, 3165-3177.

(2) Liu, J., Yang, S., Wu, W., Tian, Q., Cui, S., Dai, Z., & Jiang, C. 3D flowerlike α-Fe_2_O_3_@TiO_2_ core–shell nanostructures: general synthesis and enhanced photocatalytic performance. *ACS Sustainable Chemistry & Engineering*, **2015,***3*, 2975-2984.

(3) Jafarpour, M.; Feizpour, F.; Rezaeifard, A.; Pourmorteza, N.; Breit, B. Tandem Photocatalysis Protocol for Hydrogen Generation/Olefin Hydrogenation Using Pd-g-C_3_N_4_-Imine/TiO_2_ Nanoparticles. *Inorg. Chem.* **2021**, *60,* 9484–9495.

(4) Sun, D.; Jang, S.; Yim, S.-J.; Ye, L.; Kim, D.-P. Metal Doped Core-Shell Metal-Organic Frameworks@Covalent Organic Frameworks (MOFs@COFs) Hybrids as a Novel Photocatalytic Platform. *Adv. Funct. Mater*. **2018**, *28*, 1707110.

(5) Han, C.; Meng, P.; Waclawik, E. R.; Zhang, C.; Li, X.-H.; Yang, H.; Antonietti, M.; Xu, J. Palladium/Graphitic Carbon Nitride (g-C3N4) Stabilized Emulsion Microreactor as a Store for Hydrogen from Ammonia Borane for Use in Alkene Hydrogenation. *Angew. Chemie Int. Ed*. **2018**, *57*, 14857–14861.

(6) Sharma, P.; Sasson, Y. Sustainable Visible Light Assisted in situ Hydrogenation via a Magnesium–Water System Catalyzed by a Pd-g-C3N4 photocatalyst. ***Green Chem.***, **2019**, *21*, 261–268.

(7) Li, J.; Zhou, X.; Shang, N.; Wang, C. Nitrogen-enriched Porous Carbon Supported Pd-Nanoparticles as an Efficient Catalyst for the Transfer Hydrogenation of Alkenes. *New J. Chem.* **2018**, *42*, 16823–16828.

(8) Wan, C.; Sun, L.; Xu, L.; Cheng, D.; Chen, F.; Zhan, X.; Yang, Y. Novel NiPt Alloy Nanoparticle Decorated 2D Layered g-C3N4 Nanosheets: a Highly Efficient Catalyst for Hydrogen Generation from Hydrous Hydrazine. *J. Mater. Chem*. *A* **2019**, *7*, 8798–8804.

(9) Imran, M.; Yousaf, A. B.; Zhou, X.; Jiang, Y.-F.; Yuan, C.-Z.; Zeb, A.; Jiang, N.; Xu, A.-W. Pd/TiO Nanocatalyst with Strong Metal-Support Interaction for Highly Efficient Durable Heterogeneous Hydrogenation. *J. Phys. Chem*. *C* **2017**, *121*, 1162–1170.

(10) Liu, P.; Chen, J.; Zheng, N. Photochemical Route for Preparing Atomically Dispersed Pd1/TiO2 Catalysts on (001)-Exposed Anatase Nanocrystals and P25. *Chinese J. Catal.* **2017**, *38*, 1574–1580.

(11) Baig, R. B. N.; Verma, S.; Varma, R. S.; Nadagouda, M. N. Magnetic Fe@g-C3N4: a Photoactive Catalyst for the Hydrogenation of Alkenes and Alkynes. *ACS Sustain. Chem. Eng.* **2016**, *4*, 1661–1664.

(12) Zhang, S.; Li, J.; Xia, Z.; Wu, C.; Zhang, Z.; Ma, Y.; Qu, Y. Towards Highly Active Pd / CeO2 for Alkene Hydrogenation by Tuning Pd Dispersion and Surface Properties of the Catalysts. *Nanoscale* **2017**, *9*, 3140–3149.

(13) Liu, T.; Zeng, Y.; Zhang, H.; Wei, T.; Wu, X.; Li, N. Facile Pd-Catalyzed Chemoselective Transfer Hydrogenation of Olefins Using Formic acid in Water. *Tetrahedron Lett*. **2016**, *57*, 4845–4849.

(14) Beltram, A.; Melchionna, M.; Montini, T.; Nasi, L.; Fornasiero, P.; Prato, M. Making H_2_ from Light and Biomass-Derived Alcohols: the Outstanding Activity of Newly Designed Hierarchical MWCNT/Pd@TiO2 Hybrid Catalysts. *Green Chem*. **2017**, *19*, 2379–2389.

(15) Imamura, K.; Okubo, Y.; Ito, T.; Tanaka, A.; Hashimoto, K.; Kominami, H. Photocatalytic Hydrogenation of Alkenes to Alkanes in Alcoholic Suspensions of Palladium-loaded Titanium (iv) Oxide Without the Use of Hydrogen Gas. *RSC Adv.* **2014**, *4*, 19883–19886.

(16) Long, R.; Rao, Z.; Mao, K.; Li, Y.; Zhang, C.; Liu, Q.; Wang, C.; Li, Z.-Y.; Wu, X.; Xiong, Y. Efficient Coupling of Solar Energy to Catalytic Hydrogenation by Using Well-Designed Palladium Nanostructures. *Angew. Chemie Int. Ed.* **2015**, *54*, 2425–2430.

(17) Gong, Y.; Li, M.; Li, H.; Wang, Y. Graphitic Carbon Nitride Polymers: Promising Catalysts or Catalyst Supports for Heterogeneous Oxidation and Hydrogenation. *Green Chem.* **2015**, *17*, 715–736.

(18) Yang, S.; Gong, Y.; Zhang, J.; Zhan, L.; Ma, L.; Fang, Z.; Vajtai, R.; Wang, X.; Ajayan, P. M. Exfoliated Graphitic Carbon Nitride Nanosheets as Efficient Catalysts for Hydrogen Evolution under Visible Light. *Adv. Mater*. **2013**, *25*, 2452–2456.

(19) Chen, Z.; Sun, P.; Fan, B.; Zhang, Z.; Fang, X. In situ Template-Free Ion-Exchange Process to Prepare Visible-Light-Active g-C3N4/NiS Hybrid Photocatalysts with Enhanced Hydrogen Evolution Activity. *J. Phys. Chem*. *C* **2014**, *118*, 7801–7807.

(20) Ullah, N.; Imran, M.; Liang, K.; Yuan, C.-Z.; Zeb, A.; Jiang, N.; Qazi, U. Y.; Sahar, S.; Xu, A.-W. Highly Dispersed Ultra-Small Pd Nanoparticles on Gadolinium Hydroxide Nanorods for Efficient Hydrogenation Reactions. *Nanoscale* **2017**, *9*, 13800–13807.

(21) Ullah, N., Song, Z., Liu, W., Kuo, C. C., Ramiere, A., & Cai, X. Photo-promoted in situ reduction and stabilization of Pd nanoparticles by H_2_ at photo-insensitive Sm_2_O_3_ nanorods. *J. Colloid Interface Sci*, **2022**, *607*, 479-487.

(22) Lin, T. H., Chang, Y. H., Chiang, K. P., Wang, J. C., & Wu, M. C. Nanoscale multidimensional Pd/TiO_2_/g-C_3_N_4_ catalyst for efficient solar-driven photocatalytic hydrogen production. *Catalysts*, **2021**, *11*, 59.

(23) Sahar, K. U., Rafiq, K., Abid, M. Z., ur Rehman, U., Rauf, A., & Hussain, E. Surface sensitization of g-C_3_N­_4_/TiO_­2_ via Pd/Rb_2_­O co-catalysts: accelerating water splitting reaction for green fuel production in the absence of organic sacrificial agents. *React. Chem. Eng.,* **2023**, *8*, 2522-2536.

(24) Yavuz, C., & Ela, S. E. Fabrication of g-C_3_N_4_-reinforced CdS nanosphere-decorated TiO_2_ nanotablet composite material for photocatalytic hydrogen production and dye-sensitized solar cell application. *J. Alloys Compd*. **2023**, *936*, 168209.

(25) Chaparro-Garnica, J., Navlani-Garcia, M., Salinas-Torres, D., Morallon, E., & Cazorla-Amoros, D. Highly stable N-doped carbon-supported Pd-based catalysts prepared from biomass waste for H_2_ production from formic acid. *ACS Sustain. Chem. Eng.***2020**, *8*, 15030-15043.

(26) Zheng, T., Wu, F., Fu, H., Zeng, L., Shang, C., Zhu, L., & Guo, Z. Rational Design of Pt− Pd− Ni Trimetallic Nanocatalysts for Room‐Temperature Benzaldehyde and Styrene Hydrogenation. *Chem. Asian J.,***2021**, *16*, 2298-2306.

(27) Zhang, M., Zhao, T., Yu, C., Liu, Q., Wang, G., Yang, H., & Liu, M. Amphiphilic Pd@­micro-organohydrogels with controlled wettability for enhancing gas-liquid-solid triphasic catalytic performance. *Nano Res*., **2022**, *15*, 557-563.
